# Using virtual reality to assess gesture performance deficits in schizophrenia patients

**DOI:** 10.3389/fpsyt.2023.1191601

**Published:** 2023-06-09

**Authors:** Anastasia Pavlidou, Geoffrey Gorisse, Domna Banakou, Sebastian Walther

**Affiliations:** ^1^University of Bern, University Hospital of Psychiatry and Psychotherapy, Translation Research Centre, Bern, Switzerland; ^2^LAMPA, Arts et Métiers Institute of Technology, Changé, France; ^3^Arts and Humanities Division, New York University Abu Dhabi, Abu Dhabi, United Arab Emirates

**Keywords:** virtual reality, schizophrenia, gesture performance, social communication, social cognition

## Abstract

**Introduction:**

Gesture performance deficits are prevalent in schizophrenia patients and are strongly associated with poor social communication skills and community functioning, affecting their overall quality of life. Currently, video-recording technology is widely used in clinical settings to assess gesture production deficits in schizophrenia patients. Nevertheless, the subjective evaluation of video-recordings can encumber task assessment. The present study will aim to use virtual reality to examine its potential use as an alternative tool to objectively measure gesture performance accuracy in schizophrenia patients and healthy controls.

**Methods:**

Gesture performance in the virtual reality setting will be based on the well-established Test of Upper Limb Apraxia. Participants will be immersed in a virtual environment where they will experience themselves being embodied in a collocated virtual body seen from a first-person perspective. Motion trackers will be placed on participants' hands and elbows to track upper body movements in real-time, and to record gesture movement for later analysis. Participants will see a virtual agent sitting across from them, with a virtual table in between. The agent will perform various types of gestures and the participants' task will be to imitate those gestures as accurately as possible. Measurements from the tracking devices will be stored and analyzed to address gesture performance accuracy across groups.

**Discussion:**

This study aims to provide objective measurements of gesture performance accuracy in schizophrenia patients. If successful, the results will provide new knowledge to the gesture literature and offer the potential for novel therapeutic interventions using virtual reality technologies. Such interventions can improve gesturing and thus advance social communication skills in schizophrenia patients.

## 1. Introduction

Schizophrenia is a complex mental health disorder that affects ~1% of the general population. Schizophrenia is characterized by a wide range of clinical symptoms including disorganized thinking, paranoia, hallucinations, motor abnormalities, executive dysfunction, social withdrawal and affective flattening, all of which greatly disturb social communication processes leading to poor community functioning and quality of life (1–3). Successful social communication is highly dependent on one's ability to correctly perceive, interpret and execute socially relevant cues (4). In recent years, gestures serve as an important research tool in understanding social communication deficits in schizophrenia patients (5, 6).

Gestures are biological movements used alone or in combination with speech, to aid in social communication (7). Video-recordings of gesture performance in clinical settings show that schizophrenia patients and individuals at high-risk for psychosis tend to use hand gestures less frequently, or in the wrong context (8–10) and spend significantly less time fixating on the performed gestures (11). In addition, schizophrenia patients have deficits in correctly imitating meaningless and meaningful gestures following visual demonstration from an experimenter. Errors include slow movements with reduced amplitudes, extra movements, omissions, and body-part-as-object (12, 13). These errors are associated with impairments in postural knowledge, tool use, and social perception, suggesting a generalized impairment in non-verbal social communication in schizophrenia (14). Furthermore, deficits in gesture performance predict poor community functioning (15), and are strongly associated with symptom severity, motor abnormalities, and executive dysfunction, such as processing speed, attention, and working memory abilities (5, 11, 13, 14, 16–23).

Video recordings are easy to implement and have provided rich and notable insights on the mechanisms involved during gesture performance in schizophrenia (13, 14, 20, 24, 25). However, there are a few limitations to consider. In clinical settings, video-recordings can modify naturally occurring behaviors (26). In addition, when video-recordings are used to score participants' performance during a task, the data are categorical in nature and the process requires vigorous training for the scorer(s), which can be time consuming and cause inter-rater reliability issues. Further, the positioning of the camera can differ between sessions and participants' affecting viewpoint and body representations that can alter the evaluation of the task. The use of virtual reality (VR) technology can readily tackle most of these limitations (27).

In recent years, VR is increasingly used in the assessment and rehabilitation of movement execution (28). VR allows the creation of a virtual environment where users can interact with virtual characters via spoken language and hand gestures just as they would in the real world (29), supporting a highly immersive experience with increased ecological validity, reliability and reproducibility (30). Images of the virtual environment are displayed via the use of a head mounted display (HMD) where the users' head position and orientation are continuously tracked and updated as they move around (31, 32). Motion tracking devices can be used to substitute the users' body with a gender-matched virtual one seen from a first-person perspective and onto which their real movements are mapped in real-time, resulting in an illusionary ownership over the virtual body (33–35). Data from these tracking devices are multidimensional and provide an objective, reliable and unobtrusive measurement of real-time human behavior that surpasses that provided by video-recording data (36).

To our knowledge, no study to date has utilized VR to examine accuracy of hand gestures in schizophrenia patients. To this end, the aim of the present study is to apply VR technology to collect measurements of naturally occurring hand gestures during an imitation task in schizophrenia patients and compare their performance to healthy controls. Additionally, we will administer motor, socio-cognitive and community functioning assessments used in previous research from our lab to determine their relationship with hand gesture accuracy in VR. In accordance with previous studies (5, 13, 14, 20), we hypothesize that schizophrenia patients will perform the required gestures less accurately than healthy controls and that these deficits will be linked to their psychopathology, motor abnormalities, as well as compromised socio-cognitive and community functioning.

## 2. Materials and methods

### 2.1. Study design

This is an exploratory cross-sectional observational study using VR technology to assess the performance accuracy of different hand gestures. An HMD will be used to immerse participants in the virtual environment. Motion trackers, attached to the hands and elbows, will provide participants with real-time upper body tracking (see section VR apparatus for more details). Data from the HMD and motion trackers will be continuously stored on a computer for analyses. We plan to recruit 60 schizophrenia patients and 60 age and gender matched controls over a 2-year period. The duration of the study is ~6 h. The assessments are separated into 2-h sessions/per day and all assessments are completed within a 72-h period.

### 2.2. Participants

Schizophrenia patients will be recruited from the inpatient and outpatient departments of the University hospital of Psychiatry and Psychotherapy in Bern, Switzerland. All potential schizophrenia patients will be screened using the Mini International Neuropsychiatric Interview (37) for the DSM-5 criteria. Healthy controls will be recruited through advertisement and by word-of-mouth. Inclusion criteria for all participants include: right-handedness, 18–65 years of age, no substance abuse (except nicotine), and no history of neurological disorders or any other severe mental health disorders. Additionally, for controls only, no personal, or first-degree relatives with a history of any mental health disorders.

### 2.3. Primary outcome

#### 2.3.1. Gesture assessment in VR

The VR gesture task is based on the Test of Upper Limb Apraxia (TULIA), which was developed in accordance with the central domains and semantic characteristics of gesture performance (38, 39). TULIA measures accuracy of finger and hand movements in 48 items across two domains: the imitation domain (gesture performance following visual demonstration from the experimenter) and the pantomime domain (gesture performance following verbal command from the experimenter). Within these domains three different semantic categories of gestures exist: (a) meaningless (new gestures without semantic elements), (b) intransitive (highly learned communicative gestures, such as waving good-bye), and (c) transitive (tool-based gestures, such as using a toothbrush). Scores for each item are rated using a 0–5 scale. The maximum score earned in the TULIA test is 240 (120 per domain), which is indicative of superior gesture performance (38). Administration of the TULIA test requires both the participant and the experimenter to be seated across from each other with a table between them; both with their hands placed flat on the table. For the imitation domain, which is the focus of this study, participants are asked to execute the required gestures with their dominant hand only after the experimenter has demonstrated the gestures in a mirrored manner and has returned their hand to the original position (flat on the table). Participants are informed on the nature of the gestures following each block. The TULIA test is video-recorded and later quantified by an independent experimenter.

### 2.4. VR procedures

**Figure 1:**
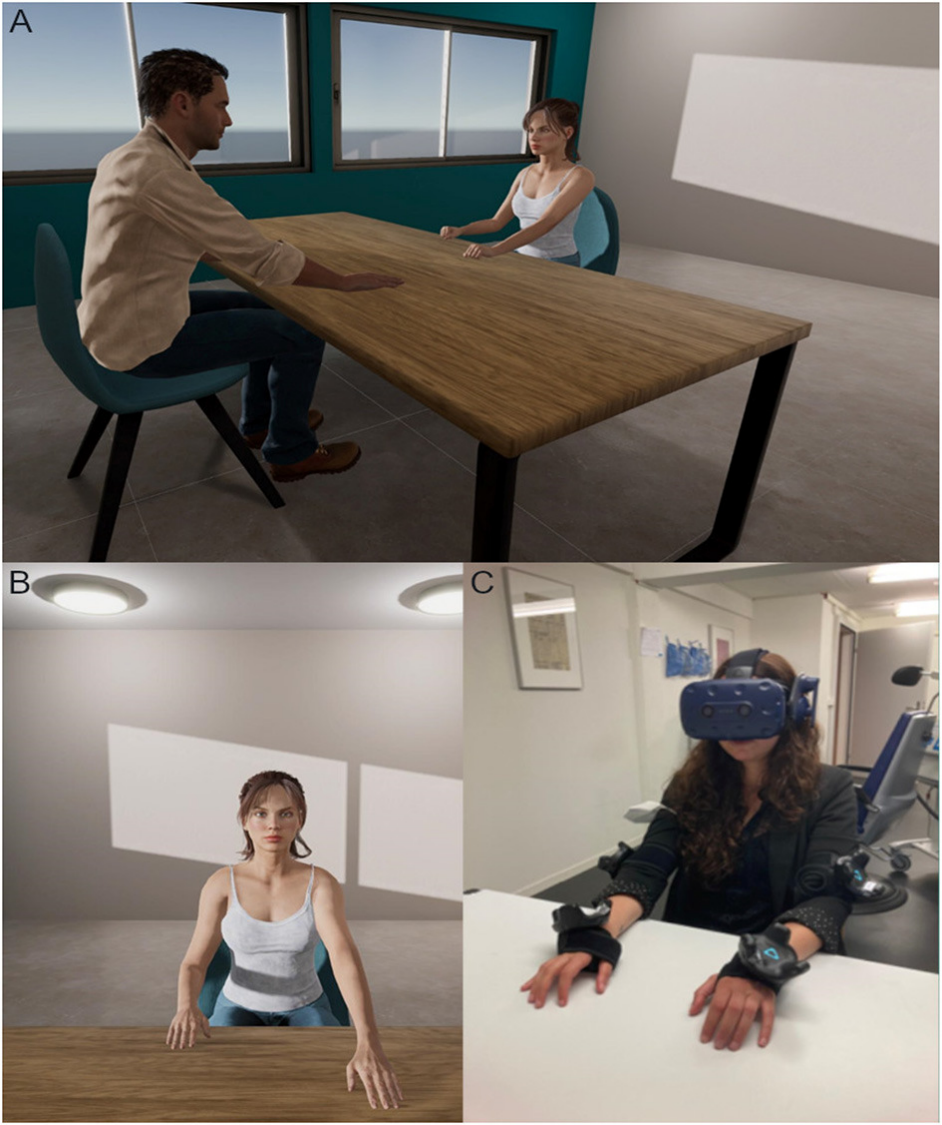
The virtual reality setup. **(A)** A third-person perspective of the virtual setup depicting the position of the participant on one side of the table and the virtual agent on the other. **(B)** The virtual environment as seen from the participant's first-person perspective. The participant has a view of their own hands and gender-matched body and they can see the virtual agent sitting across from them with the hands resting on the table. **(C)** The participant in the physical laboratory equipped with the HMD and upper body tracking devices.

The development of the virtual environment for gesture assessment in VR was inspired from clinical applications of the TULIA task and created using the real-time 3D engine Unity 2020 LTS.^1^ Animations for the TULIA movements were recorded using the Glycon 3D^2^ motion capture software and edited with Autodesk MotionBuilder 2019.^3^ The virtual environment consists of an office room equipped with a table in its center (Figure 1A). Windows and a door are also present on the left and right side of the room, respectively. Across from participants, on the other side of the table, a virtual agent is seated on a chair with her hands flat on the table (Figure 1B). Outside the virtual environment, in the physical laboratory, participants are placed in a similar scenario with their hands flat on a table in front of them. Motion trackers are placed on the hands and elbows of each arm, and the HMD is adjusted for each participant (Figure 1C). The HMD provides a stereoscopic display of the virtual environment and of the autonomous agent seen from an embodied first-person perspective, while the motion trackers provide participants with virtual arms that they can control using their own movements. Participants' virtual body will be gender matched. Once immersed in the virtual environment participants will be instructed via pre-recorded verbal instructions to look around and describe what they see. In addition, they will be instructed to move their hands up and down to induce the illusion of body ownership over the virtual body, following earlier examples (33–35). After this introductory phase, participants will be trained on the task. They will be informed to closely look at the movements of the agent seated across from them performing the gestures in a mirrored manner. Their task will be to imitate those same gestures as accurately as possible using their dominant hand (right hand). To ensure that participants perform the gestures only after the agent places her hands back on the table, a red light is added as a visual cue, and participants will be informed to only perform the gestures once this light turns off. Similar to the TULIA task, participants will be informed on the nature of the gestures before each block. Each block contains 6 different gestures and will be repeated twice during the experiment. All gesture blocks (meaningless, intransitive, and transitive) will be randomized across participants. A post-experiment questionnaire will be used to record participants' subjective feeling of presence and embodiment with respect to their virtual body, as well as, other questions related to how participants evaluate the virtual female agent and their interaction, and overall experience using a −3 (“not at all”) to a 3 (“very much”) scale (Table 1). In addition, possible side effects (i.e., dizziness), as well as, personal thoughts and feelings regarding participants' exposure to VR will also be accounted for directly after the experiment and at the follow-up session, 2-weeks after exposure. The VR procedures are demonstrated in Supplementary Movie S1.

**Table 1:** The post-VR questionnaire.

**Variable**	**Question**
Body	“How much did you feel that the *virtual body* you saw when looking down at yourself was your own body?” *(−3…3)*
Mirror	“How much did you feel that the *virtual body* you saw when looking at yourself in the *mirror* was your own body?” *(−3…3)*
Agency	“How much did you feel that the movements of the virtual body were caused by your own movements?” *(−3…3)*
Real person	“How much did you feel like the person sitting in front of you was a real person?” *(−3…3)*
Friendly person	“Was the virtual person sitting in front of you friendly toward you?” *(−3…3)*
Trustworthy	“Did you find the virtual person sitting in front of you trustworthy?” *(−3…3)*
Distress	“Did the virtual person sitting in front of you make you feel distressed?” *(−3…3)*
Valence	Please circle the manikin that you think better expresses how you felt while being in the virtual room. Choose one manikin in each of the two figures.
	*Valence manikin*
Arousal	Please circle the manikin that you think better expresses how you felt while being in the virtual room. Choose one manikin in each of the two figures.
	*Arousal manikin*

### 2.5. VR apparatus

The HTC VIVE Pro^4^ virtual reality headset will be used to display the virtual environment. It has a combined resolution of 2,880 × 1,600 pixels (1,440 × 1,600 pixels per eye), a refresh rate of 90 Hz, and a field of view of 110°. The motion tracking equipment which will allow participants to control their virtual body arms in real time is performed using the VIVE trackers (four in total−2 placed on the hands and 2 on the elbows as seen in Figure 1C). The application will run on a computer with a Core i9-11900H @ 3.50 processor and an Nvidia GeForce RTX 3080 graphics card.

### 2.6. Secondary outcomes

We will include additional non-verbal communication tasks outside of VR to assess participants' ability to recognize and perceive non-verbal social cues such facial expressions, biological movements, and voice tone. Motor, cognitive and community functioning domains will be assessed using well-established standardized rating scales and symptom severity and antipsychotic medication usage in all patients will also be accounted for. All clinical, motor, socio-cognitive and community functioning assessments can be found in Table 2.

**Table 2:** Clinical, motor, socio-cognitive and community functioning assessments of participants.

**Secondary outcomes**	**References**
**Psychopathology**	
Brief negative symptom scale	(40)
Positive and negative syndrome scale	(41)
Thought and language disorder scale	(42)
**Motor scales**	
Abnormal involuntary movement scale	(43)
Bush Francis Catatonia rating scale	(44)
Neurological evaluation scale	(45)
Salpêtrière retardation rating scale	(46)
Unified Parkinson's disease rating scale	(47)
**Socio-cognitive assessments**	
Biological motion	(48, 49)
Dot counting task	(50, 51)
EMOREC-B	(52)
Hinting task	(53)
MATRICS consensus cognitive battery	(54)
Postural knowledge test	(55)
Profile of nonverbal sensitivity	(56)
**Community functioning**	
Global assessment of functioning	(57)
Social level of functioning	(58)
Social and occupational functioning assessment score	(59)
University of California, San Diego performance-based skills assessment brief	(60)
**Self-reports**	
Brief assessment of gestures	(61)
Post-VR questionnaire on presence and embodiment	(62)
Self-report of negative symptoms	(63)

## 3. Statistical analysis

Measurements collected from the tracking devices during gesture performance will be analyzed using linear mixed models. These will include upper body rotational data per gesture across all experimental blocks. Data are saved at a frequency of 60 Hz (16 ms). The rotational data between participants and the virtual agent performing the gestures will be compared across time based on distance metrics such as Dynamic Time Warping (DTW) that measures similarity between time series by considering the temporal relationship between rotational vectors. Group (patients and healthy controls) and gesture category (meaningless, intransitive, and transitive), as well as, their interaction will be added as fixed effects, while a single intercept parameter estimated for each participant will be added as random effects. Covariates in the model will also be considered if demographic characteristics such as age, gender or education are different between patients and controls. In addition, differences in symptom severity and motor deficits between inpatient and outpatient participants will also be considered. The Kruskal–Wallis test will be used to assess such differences. In addition, we will apply partial correlation analyses using the spearman method to examine the relationship between gesture performance accuracy in VR with symptom severity, motor, socio-cognitive and community functioning scores, while controlling for medication. This will help in determining if gesture performance accuracy using VR in patients is linked to a specific domain or is generalized across multiple domains.

## 4. Trial status

Recruitment for the study begun November 1st 2022, and is expected to be completed November 2024. As of May 2023, we recruited 23 patients and 8 controls, and completed assessments for 19 patients and 7 controls.

## 5. Discussion

The crucial feature of this study is the implementation of a VR setup to assess real-time movements to objectively quantify gesture performance accuracy in schizophrenia patients and healthy controls. To date, assessment of gesture performance in schizophrenia patients is mostly based on categorical data, and doesn't take into account all the attributes and features associated with gesture performance. VR captures multidimensional data that may reveal hidden insights on the mechanisms attributed to gesture deficits in schizophrenia patients. If our VR setup proves to be successful in capturing and measuring gesture deficits in schizophrenia patients this has the potential to generate new knowledge regarding non-verbal communication that can help in designing therapeutic interventions to improve treatments for schizophrenia patients (36). Such treatments are valuable as there are often substantial costs to patients, their family and friends, caregivers and to the society as a whole (64). In the following section, we briefly discuss the types of therapeutic interventions available to schizophrenia patients, and discuss future directions on how VR can support and expand their impact.

Thus far, most treatments for schizophrenia heavily relied on pharmacological interventions, with heterogeneous results (23). Recent evidence suggests that neurostimulation (65–67), cognitive remediation therapy (52, 68–70) and social skills training (70, 71) promote neuroplasticity and can alleviate non-verbal communication deficits in schizophrenia patients, such as the evaluation and interpretation of emotional expressions, eye contact and body language but none are exclusive in tackling gesture deficits. In fact, therapeutic interventions specifically targeting gesture deficits and, in particular, gesture performance deficits in schizophrenia are limited. One recent study used a specific multimodal speech-gesture training that integrates non-verbal communication, working memory abilities and natural communication (72) to improve gesture processing in schizophrenia and showed significant improvements in patients' quality of life, which was significantly correlated with changes in the neural processing of abstract speech-gesture content (73). Another study, which combines neurostimulation and social cognitive remediation therapy to examine their effectiveness in improving gesture performance in schizophrenia is currently underway (68).

While conventional therapeutic interventions are essential in recovery, they are organized in accordance to one's environment, reaching only a portion of the patients who can benefit, and are strongly dependent on the availability and expertise of therapists. The application of VR in clinical settings can provide flexible, ecologically valid, reproducible and highly-controlled environments of real-life situations offering cost-effective exposure therapy in terms of time and expenses while reaching a much larger population. This coupled with the convenience and privacy it provides, clinicians have utilized VR in pain management, cognitive and motor rehabilitation in stroke and Parkinson's patients, and therapy and training exposure for patients suffering from anxiety, depression and psychosis (74–76).

VR use in schizophrenia has been successfully applied to alleviate auditory hallucinations and paranoid ideation (77, 78) to improve interpersonal communication by having patients interact with various characters with different behaviors, and help them acquire new social and interview training skills (79, 80). Patients in such treatments have previously expressed enjoyment and increased motivation when using VR (79, 81). However, no previous studies have explored the potential of VR in movement rehabilitation in schizophrenia patients in order to improve gesture performance. Evidence from neurological patients suggests that VR as a multifaceted system promotes motor learning, a process where the acquisition of newly learned movement skills via experience and practice induces a permanent change in one's ability to execute these newly learned movements (82). If our VR setup proves effective in measuring movement discrepancies during gesture performance in schizophrenia patients, it can be applied to generate new therapeutic interventions, specifically targeting gesture deficits using motor learning, and assess potential changes over time resulting from these interventions. Moreover, VR's adaptable and diverse technological features enable its integration with existing therapies like cognitive remediation therapy and social skills training. This integration can provide more accurate and objective measurements of naturalistic behaviors arising from ongoing interactions that cannot otherwise be quantified using traditional methods. This constitutes VR the ideal candidate in providing patients with an enjoyable and add-on alternative to help in retaining and transferring motor learning in their daily life (83, 84). These newly acquired movement skills can improve how schizophrenia patients express themselves to their family, friends', doctor, or therapist, and how they handle day-to-day tasks, thus enhancing their overall social communication and functioning, whilst promoting independence.

## 6. Limitations

The study does have certain limitations. First, this is a cross-sectional study measuring gesture performance accuracy in VR at a single time-point. Conducting longitudinal studies could offer a more comprehensive understanding of how gesture performance can progress over time in schizophrenia patients. Second, it is crucial to take into account the potential effects of antipsychotic medication on gesture performance. While we intend to account for antipsychotic medication use as a covariate in our analyses, it is important to acknowledge that patients might be on other types of medication that may still influence our findings. Third, although the optical body tracking was designed to avoid occlusion and reduce tracking discrepancies, tracking losses might still occur leading to potential inconsistencies in the data. Fourth, our application currently supports only gender-matched virtual bodies to substitute the participants' real body. However, in future updates, we aim to use personalized avatars that can consider ethnic characteristics as well. This enhancement could allow for accommodating potential variations in body ownership. This may be particularly important to address in patients with schizophrenia, whereas for healthy individuals it has been demonstrated that body ownership illusions can be induced over distinct virtual bodies, including different race (85–87).

## 7. Conclusion

The study aims to provide an objective measurement in assessing gesture performance accuracy in schizophrenia patients and healthy controls using VR. If this study proves to be successful it opens the possibility of utilizing VR paradigms to carry-out therapeutic interventions using motor learning elements to improve gesture deficits in schizophrenia patients and measure possible changes over time in response to these interventions. These types of interventions can be beneficial to other mental health disorders patients who exhibit gesture deficits such as depression (88, 89) or suffer from social cognitive deficits that interfere with non-verbal communication skills such as bipolar and autism spectrum disorders (90, 91).

## Ethics statement

The studies involving human participants were reviewed and approved by Kantonale Ethikkommision Bern (KEK 2021-02047). The patients/participants provided their written informed consent to participate in this study.

## Author contributions

AP: conceptualization, organization, obtained ethics approval, supervision, and drafted the manuscript. GG: designed and set up the VR environment and apparatus. DB: designed and set up the VR environment and apparatus and funding. SW: funding and supervision. All authors reviewed and edited the manuscript. All authors contributed to the article and approved the submitted version.

## Footnotes

^1^ https://unity.com/

^2^ https://www.glycon3d.com/

^3^ https://www.autodesk.com/products/motionbuilder

^4^ https://www.vive.com/us/product/vive-pro2-full-kit/overview/

## References

[1] Green MF Horan WP Lee J. Social cognition in schizophrenia. Nat Rev Neurosci. (2015) 16:620–31. 10.1038/nrn4005 26373471

[2] McCutcheon RA Marques TR Howes OD. Schizophrenia—an overview. JAMA Psychiatry. (2020) 77:201–10. 10.1001/jamapsychiatry.2019.3360 31664453

[3] Wible CG. Schizophrenia as a disorder of social communication. Schizophr Res Treat. (2012) 2012:920485. 10.1155/2012/920485 22966453 PMC3420370

[4] Frith CD Frith U. Social cognition in humans. Curr Biol. (2007) 17:R724–32. 10.1016/j.cub.2007.05.068 17714666

[5] Pavlidou A Chapellier V Maderthaner L von Kanel S Walther S. Using dynamic point light display stimuli to assess gesture deficits in schizophrenia. Schizophr Res Cogn. (2022) 28:100240. 10.1016/j.scog.2022.100240 35242609 PMC8866720

[6] Walther S Mittal VA. Why we should take a closer look at gestures. Schizophr Bull. (2016) 42:259–61. 10.1093/schbul/sbv229 26773476 PMC4753618

[7] Goldin-Meadow S Alibali MW. Gesture's role in speaking, learning, creating language. Annu Rev Psychol. (2013) 64:257–83. 10.1146/annurev-psych-113011-143802 22830562 PMC3642279

[8] Lavelle M Dimic S Wildgrube C McCabe R Priebe S. Non-verbal communication in meetings of psychiatrists and patients with schizophrenia. Acta Psychiatr Scand. (2015) 131:197–205. 10.1111/acps.12319 25124849

[9] Lavelle M Healey PG McCabe R. Is nonverbal communication disrupted in interactions involving patients with schizophrenia? Schizophr Bull. (2013) 39:1150–8. 10.1093/schbul/sbs091 22941744 PMC3756773

[10] Millman ZB Goss J Schiffman J Mejias J Gupta T Mittal VA. Mismatch and lexical retrieval gestures are associated with visual information processing, verbal production, and symptomatology in youth at high risk for psychosis. Schizophr Res. (2014) 158:64–8. 10.1016/j.schres.2014.06.007 25000911 PMC4152422

[11] Gupta T Osborne KJ Mittal VA. Abnormal gesture perception and clinical high-risk for psychosis. Schizophr Bull. (2021) 47:938–47. 10.1093/schbul/sbab056 33963750 PMC8266619

[12] Walther S Mittal VA Stegmayer K Bohlhalter S. Gesture deficits and apraxia in schizophrenia. Cortex. (2020) 133:65–75. 10.1016/j.cortex.2020.09.017 33099076 PMC7945048

[13] Walther S Vanbellingen T Muri R Strik W Bohlhalter S. Impaired gesture performance in schizophrenia. particular vulnerability of meaningless pantomimes. Neuropsychologia. (2013) 51:2674–8. 10.1016/j.neuropsychologia.2013.08.017 24001392

[14] Walther S Stegmayer K Sulzbacher J Vanbellingen T Muri R Strik W . Nonverbal social communication and gesture control in schizophrenia. Schizophr Bull. (2015) 41:338–45. 10.1093/schbul/sbu222 25646526 PMC4332963

[15] Walther S Eisenhardt S Bohlhalter S Vanbellingen T Muri R Strik W . Gesture performance in schizophrenia predicts functional outcome after 6 months. Schizophr Bull. (2016) 42:1326–33. 10.1093/schbul/sbw124 27566843 PMC5049539

[16] Dutschke LL Stegmayer K Ramseyer F Bohlhalter S Vanbellingen T Strik W . Gesture impairments in schizophrenia are linked to increased movement and prolonged motor planning and execution. Schizophr Res. (2018) 200:42–9. 10.1016/j.schres.2017.07.012 28709771

[17] Matthews N Gold BJ Sekuler R Park S. Gesture imitation in schizophrenia. Schizophr Bull. (2013) 39:94–101. 10.1093/schbul/sbr062 21765171 PMC3523902

[18] Nagels A Kircher T Grosvald M Steines M Straube B. Evidence for gesture-speech mismatch detection impairments in schizophrenia. Psychiatry Res. (2019) 273:15–21. 10.1016/j.psychres.2018.12.107 30639559

[19] Park S Matthews N Gibson C. Imitation, simulation, and schizophrenia. Schizophr Bull. (2008) 34:698–707. 10.1093/schbul/sbn048 18499703 PMC2632442

[20] Walther S Vanbellingen T Muri R Strik W Bohlhalter S. Impaired pantomime in schizophrenia: association with frontal lobe function. Cortex. (2013) 49:520–7. 10.1016/j.cortex.2011.12.008 22264446

[21] Chapellier V Pavlidou A Maderthaner L von Kanel S Walther S. The impact of poor nonverbal social perception on functional capacity in schizophrenia. Front Psychol. (2022) 13:804093. 10.3389/fpsyg.2022.804093 35282219 PMC8904900

[22] Corcoran CM Mittal VA Bearden CE Gur RE Hitczenko K Bilgrami Z . Language as a biomarker for psychosis: a natural language processing approach. Schizophr Res. (2020) 226:158–66. 10.1016/j.schres.2020.04.032 32499162 PMC7704556

[23] Wüthrich F Pavlidou A Stegmayer K Eisenhardt S Moor J Schäppi L . Nonverbal communication remains untouched: no beneficial effect of symptomatic improvement on poor gesture performance in schizophrenia. Schizophr Res. (2020) 223:258–64. 10.1016/j.schres.2020.08.013 32883557 PMC7952214

[24] Straube B Green A Sass K Kirner-Veselinovic A Kircher T. Neural integration of speech and gesture in schizophrenia: evidence for differential processing of metaphoric gestures. Hum Brain Mapp. (2013) 34:1696–712. 10.1002/hbm.22015 22378493 PMC6870001

[25] Choudhury M Steines M Nagels A Riedl L Kircher T Straube B. Neural basis of speech-gesture mismatch detection in schizophrenia spectrum disorders. Schizophr Bull. (2021) 47:1761–71. 10.1093/schbul/sbab059 34050672 PMC8530401

[26] Haidet KK Tate J Divirgilio-Thomas D Kolanowski A Happ MB. Methods to improve reliability of video-recorded behavioral data. Res Nurs Health. (2009) 32:465–74. 10.1002/nur.20334 19434651 PMC2713814

[27] van Harten PN Walther S Kent JS Sponheim SR Mittal VA. The clinical and prognostic value of motor abnormalities in psychosis, and the importance of instrumental assessment. Neurosci Biobehav Rev. (2017) 80:476–87. 10.1016/j.neubiorev.2017.06.007 28711662

[28] Perez-Marcos D Bieler-Aeschlimann M Serino A. Virtual reality as a vehicle to empower motor-cognitive neurorehabilitation. Front Psychol. (2018) 9:2120. 10.3389/fpsyg.2018.02120 30450069 PMC6224455

[29] Perez-Marcos D Solazzi M Steptoe W Oyekoya O Frisoli A Weyrich T . A fully immersive set-up for remote interaction and neurorehabilitation based on virtual body ownership. Front Neurol. (2012) 3:110. 10.3389/fneur.2012.00110 22787454 PMC3392697

[30] Parsons TD Phillips AS. Virtual reality for psychological assessment in clinical practice. Pract Innov. (2016) 1:197. 10.1037/pri0000028

[31] Sanchez-Vives MV Slater M. From presence to consciousness through virtual reality. Nat Rev Neurosci. (2005) 6:332–39. 10.1038/nrn1651 15803164

[32] Spanlang B Normand J-M Borland D Kilteni K Giannopoulos E Pomés A . How to build an embodiment lab: achieving body representation illusions in virtual reality. Front Robot AI. (2014) 1:9. 10.3389/frobt.2014.00009

[33] Kilteni K Groten R Slater M. The sense of embodiment in virtual reality. PRESENCE Teleoperat Virtual Environ. (2012) 21:373–87. 10.1162/PRES_a_00124

[34] Maselli A Slater M. The building blocks of the full body ownership illusion. Front Hum Neurosci. (2013) 7:83. 10.3389/fnhum.2013.00083 23519597 PMC3604638

[35] Slater M Spanlang B Sanchez-Vives MV Blanke O. First person experience of body transfer in virtual reality. PLoS ONE. (2010) 5:e10564. 10.1371/journal.pone.0010564 20485681 PMC2868878

[36] Freeman D Reeve S Robinson A Ehlers A Clark D Spanlang B . Virtual reality in the assessment, understanding, and treatment of mental health disorders. Psychol Med. (2017) 47:2393–400. 10.1017/S003329171700040X 28325167 PMC5964457

[37] Sheehan DV Lecrubier Y Sheehan KH Amorim P Janavs J Weiller E . The Mini-International Neuropsychiatric Interview (M.I.N.I.): the development and validation of a structured diagnostic psychiatric interview for DSM-IV and ICD-10. J Clin Psychiatry. (1998) 59(Suppl. 20):22–33. 9881538

[38] Vanbellingen T Kersten B Van Hemelrijk B Van De Winckel A Bertschi M Müri R . Comprehensive assessment of gesture production: a new test of upper limb apraxia (TULIA). Eur J Neurol. (2010) 17:59–66. 10.1111/j.1468-1331.2009.02741.x 19614961

[39] Bachofner H Scherer KA Vanbellingen T Bohlhalter S Stegmayer K Walther S. Validation of the Apraxia Screen TULIA (AST) in Schizophrenia. Neuropsychobiology. (2022) 81:311–21. 10.1159/000523778 35367989 PMC9533426

[40] Kirkpatrick B Strauss GP Nguyen L Fischer BA Daniel DG Cienfuegos A . The brief negative symptom scale: psychometric properties. Schizophr Bull. (2011) 37:300–5. 10.1093/schbul/sbq059 20558531 PMC3044634

[41] Kay SR Fiszbein A Opler LA. The positive and negative syndrome scale (PANSS) for schizophrenia. Schizophr Bull. (1987) 13:261–76. 10.1093/schbul/13.2.261 3616518

[42] Kircher T Krug A Stratmann M Ghazi S Schales C Frauenheim M . A rating scale for the assessment of objective and subjective formal Thought and Language Disorder (TALD). Schizophr Res. (2014) 160:216–21. 10.1016/j.schres.2014.10.024 25458572

[43] Guy W. ECDEU Assessment Manual for Psychopharmacology. Rockville, MD: US Department of Health, Education, and Welfare, Public Health Service (1976). 10.1037/e591322011-001

[44] Bush G Fink M Petrides G Dowling F Francis A. Catatonia. I. Rating scale and standardized examination. Acta Psychiatr Scand. (1996) 93:129–36. 10.1111/j.1600-0447.1996.tb09814.x 8686483

[45] Buchanan RW Heinrichs DW. The Neurological Evaluation Scale (NES): a structured instrument for the assessment of neurological signs in schizophrenia. Psychiatry Res. (1989) 27:335–50. 10.1016/0165-1781(89)90148-0 2710870

[46] Dantchev N Widlocher DJ. The measurement of retardation in depression. J Clin Psychiatry. (1998) 59:19–25. 9818627

[47] S. Fahn. Unified Parkinson's disease rating scale. Recent Dev Parkinsons Dis. (1987) 18:738–50.

[48] Pavlidou A Schnitzler A Lange J. Distinct spatio-temporal profiles of beta-oscillations within visual and sensorimotor areas during action recognition as revealed by ME. Cortex. (2014) 54:106–16. 10.1016/j.cortex.2014.02.007 24657479

[49] Pavlidou A Schnitzler A Lange J. Interactions between visual and motor areas during the recognition of plausible actions as revealed by magnetoencephalography. Hum Brain Mapp. (2014) 35:581–92. 10.1002/hbm.22207 23117670 PMC6869263

[50] Pavlidou A Gallagher M Lopez C Ferre ER. Let's share our perspectives, but only if our body postures match. Cortex. (2019) 119:575–9. 10.1016/j.cortex.2019.02.019 30910224

[51] Samson D Apperly IA Braithwaite JJ Andrews BJ Bodley Scott ES. Seeing it their way: evidence for rapid and involuntary computation of what other people see. J Exp Psychol Hum Percept Perform. (2010) 36:1255. 10.1037/a0018729 20731512

[52] Mueller DR Schmidt SJ Roder V. One-year randomized controlled trial and follow-up of integrated neurocognitive therapy for schizophrenia outpatients. Schizophr Bull. (2015) 41:604–16. 10.1093/schbul/sbu223 25713462 PMC4393700

[53] Corcoran R Mercer G Frith CD. Schizophrenia, symptomatology and social inference: investigating “theory of mind” in people with schizophrenia. Schizophr Res.(1995) 17:5–13. 10.1016/0920-9964(95)00024-G 8541250

[54] Nuechterlein KH Green MF Kern RS Baade LE Barch DM Cohen JD . The MATRICS Consensus Cognitive Battery, part 1: test selection, reliability, and validity. Am J Psychiatry. (2008) 165:203–13. 10.1176/appi.ajp.2007.07010042 18172019

[55] Mozaz M Rothi LJG Anderson JM Crucian GP Heilman KM. Postural knowledge of transitive pantomimes and intransitive gestures. J Int Neuropsychol Soc. (2002) 8:958–62. 10.1017/S1355617702870114 12405548

[56] R. Rosenthal. Sensitivity to Nonverbal Communication: The PONS Test. Baltimore, MD: Johns Hopkins University Press (1978). 10.1016/B978-0-12-761350-5.50012-4

[57] Endicott J Spitzer RL Fleiss JL Cohen J. The global assessment scale: a procedure for measuring overall severity of psychiatric disturbance. Arch Gen Psychiatry. (1976) 33:766–71. 10.1001/archpsyc.1976.01770060086012 938196

[58] Schneider LC Struening EL. SLOF: a behavioral rating scale for assessing the mentally ill. Soc Work Res Abstr. (1983) 19:9–21. 10.1093/swra/19.3.9 10264257

[59] Morosini PL Magliano L Brambilla LA Ugolini S Pioli R. Development, reliability and acceptability of a new version of the DSM-IV Social and Occupational Functioning Assessment Scale (SOFAS) to assess routine social funtioning. Acta Psychiatr Scand. (2000) 101:323–9. 10.1034/j.1600-0447.2000.101004323.x 10782554

[60] Mausbach BT Depp CA Bowie CR Harvey PD McGrath JA Thronquist MH . Sensitivity and specificity of the UCSD Performance-based Skills Assessment (UPSA-B) for identifying functional milestones in schizophrenia. Schizophr Res. (2011) 132:165–70. 10.1016/j.schres.2011.07.022 21843926 PMC3195873

[61] Nagels A Kircher T Steines M Grosvald M Straube B. A brief self-rating scale for the assessment of individual differences in gesture perception and production. Learn Individ Diff. (2015) 39:73–80. 10.1016/j.lindif.2015.03.008

[62] Banakou D Slater M. Body ownership causes illusory self-attribution of speaking and influences subsequent real speaking. Proc Natl Acad Sci USA. (2014) 111:17678–83. 10.1073/pnas.1414936111 25422444 PMC4267370

[63] Dollfus S Mach C Morello R. Self-evaluation of negative symptoms ^*^ : a novel tool to assess negative symptoms. Schizophr Bull. (2015) 42:571–8. 10.1093/schbul/sbv161 26564898 PMC4838089

[64] Kotzeva A Mittal D Desai S Judge D Samanta K. Socioeconomic burden of schizophrenia: a targeted literature review of types of costs and associated drivers across 10 countries. J Med Econ. (2023) 26:70–83. 10.1080/13696998.2022.2157596 36503357

[65] Vanbellingen T Pastore-Wapp M Kubel S Nyffeler T Schupfer AC Kiefer C . Interhemispheric facilitation of gesturing: a combined theta burst stimulation and diffusion tensor imaging study. Brain Stimul. (2020) 13:457–63. 10.1016/j.brs.2019.12.013 31911072

[66] Walther S Kunz M Muller M Zurcher C Vladimirova I Bachofner H . Single session transcranial magnetic stimulation ameliorates hand gesture deficits in schizophrenia. Schizophr Bull. (2020) 46:286–93. 10.1093/schbul/sbz078 31634401 PMC7442336

[67] Walther S Alexaki D Schoretsanitis G Weiss F Vladimirova I Stegmayer K . Inhibitory repetitive transcranial magnetic stimulation to treat psychomotor slowing: a transdiagnostic, mechanism-based randomized double-blind controlled trial. Schizophr Bull Open. (2020) 1:sgaa020. 10.1093/schizbullopen/sgaa020

[68] Chapellier V Pavlidou A Mueller DR Walther S. Brain stimulation and group therapy to improve gesture and social skills in schizophrenia-the study protocol of a randomized, sham-controlled, three-arm, double-blind trial. Front Psychiatry. (2022) 13:909703. 10.3389/fpsyt.2022.909703 35873264 PMC9301234

[69] Mueller DR Roder V. Integrated psychological therapy for schizophrenia patients. Expert Rev Neurother. (2007) 7:1–3. 10.1586/14737175.7.1.1 17187489

[70] Morin L Franck N. Rehabilitation interventions to promote recovery from schizophrenia: a systematic review. Front Psychiatry. (2017) 8:100. 10.3389/fpsyt.2017.00100 28659832 PMC5467004

[71] Kopelowicz A Liberman RP Zarate R. Recent advances in social skills training for schizophrenia. Schizophr Bull. (2006) 32:S12–23. 10.1093/schbul/sbl023 16885207 PMC2632540

[72] Riedl L Nagels A Sammer G Straube B. A multimodal speech-gesture training intervention for patients with schizophrenia and its neural underpinnings – the study protocol of a randomized controlled pilot trial. Front Psychiatry. (2020) 11:110. 10.3389/fpsyt.2020.00110 32210849 PMC7068208

[73] Riedl L Nagels A Sammer G Choudhury M Nonnenmann A Sütterlin A . Multimodal speech-gesture training in patients with schizophrenia spectrum disorder: effects on quality of life and neural processing. Schizophr Res. (2022) 246:112–25. 10.1016/j.schres.2022.06.009 35759877

[74] Cieślik B Mazurek J Rutkowski S Kiper P Turolla A Szczepańska-Gieracha J. Virtual reality in psychiatric disorders: a systematic review of reviews. Complement Ther Med. (2020) 52:102480. 10.1016/j.ctim.2020.102480 32951730

[75] Goudman L Jansen J Billot M Vets N De Smedt A Roulaud M . Virtual reality applications in chronic pain management: systematic review and meta-analysis. JMIR Serious Games. (2022) 10:e34402. 10.2196/34402 35536641 PMC9131143

[76] Tieri G Morone G Paolucci S Iosa M. Virtual reality in cognitive and motor rehabilitation: facts, fiction and fallacies. Expert Rev Med Devices. (2018) 15:107–17. 10.1080/17434440.2018.1425613 29313388

[77] du Sert OP Potvin S Lipp O Dellazizzo L Laurelli M Breton R . Virtual reality therapy for refractory auditory verbal hallucinations in schizophrenia: a pilot clinical trial. Schizophr Res. (2018) 197:176–81. 10.1016/j.schres.2018.02.031 29486956

[78] Moritz S Voigt M Köther U Leighton L Kjahili B Babur Z . Can virtual reality reduce reality distortion? Impact of performance feedback on symptom change in schizophrenia patients. J Behav Ther Exp Psychiatry. (2014) 45:267–71. 10.1016/j.jbtep.2013.11.005 24384509

[79] Rus-Calafell M Garety P Sason E Craig TJ Valmaggia LR. Virtual reality in the assessment and treatment of psychosis: a systematic review of its utility, acceptability and effectiveness. Psychol Med. (2018) 48:362–91. 10.1017/S0033291717001945 28735593

[80] Valmaggia LR Day F Rus-Calafell M. Using virtual reality to investigate psychological processes and mechanisms associated with the onset and maintenance of psychosis: a systematic review. Soc Psychiatry Psychiatr Epidemiol. (2016) 51:921–36. 10.1007/s00127-016-1245-0 27262562

[81] Adery LH Ichinose M Torregrossa LJ Wade J Nichols H Bekele E . The acceptability and feasibility of a novel virtual reality based social skills training game for schizophrenia: preliminary findings. Psychiatry Res. (2018) 270:496–502. 10.1016/j.psychres.2018.10.014 30326433 PMC6314809

[82] Schmidt RA Lee TD Winstein C Wulf G Zelaznik HN. Motor control and learning: a behavioral emphasis. Hum Kinet. (2018).

[83] Levac DE Sveistrup H. Motor learning and virtual reality. In:Weiss PL Keshner EA Levin MF, editors. Virtual Reality for Physical and Motor Rehabilitation. New York, NY: Springer New York (2014). 10.1007/978-1-4939-0968-1_3

[84] Pavlidou A Walther S. Using virtual reality as a tool in the rehabilitation of movement abnormalities in schizophrenia. Front Psychol. (2020) 11:607312. 10.3389/fpsyg.2020.607312 33488466 PMC7817610

[85] Banakou D Beacco A Neyret S Blasco-Oliver M Seinfeld S Slater M. Virtual body ownership and its consequences for implicit racial bias are dependent on social context. R Soc Open Sci. (2020) 7:201848. 10.1098/rsos.201848 33489296 PMC7813259

[86] Banakou D Hanumanthu PD Slater M. Virtual embodiment of white people in a black virtual body leads to a sustained reduction in their implicit racial bias. Front Hum Neurosci. (2016) 10:601. 10.3389/fnhum.2016.00601 27965555 PMC5126081

[87] Peck TC Seinfeld S Aglioti SM Slater M. Putting yourself in the skin of a black avatar reduces implicit racial bias. Conscious Cogn. (2013) 22:779–87. 10.1016/j.concog.2013.04.016 23727712

[88] Pavlidou A Viher PV Bachofner H Weiss F Stegmayer K Shankman SA . Hand gesture performance is impaired in major depressive disorder: a matter of working memory performance? J Affect Disord. (2021) 292:81–8. 10.1016/j.jad.2021.05.055 34107424 PMC8797922

[89] Suffel A Nagels A Steines M Kircher T Straube B. Feeling addressed! The neural processing of social communicative cues in patients with major depression. Hum Brain Mapp. (2020) 41:3541–54. 10.1002/hbm.25027 32432387 PMC7416026

[90] Gillissie ES Lui LMW Ceban F Miskowiak K Gok S Cao B . Deficits of social cognition in bipolar disorder: systematic review and meta-analysis. Bipolar Disord. (2022) 24:137–48. 10.1111/bdi.13163 34825440

[91] Pagni BA Walsh MJM Rogers C Braden BB. Social cognition in autism spectrum disorder across the adult lifespan: influence of age and sex on reading the mind in the eyes task in a cross-sectional sample. Front Integr Neurosci. (2020) 14:571408. 10.3389/fnint.2020.571408 33013336 PMC7498724

